# Targeted sequencing of selected functional genes in patients with wild-type transthyretin amyloidosis

**DOI:** 10.1186/s13104-023-06491-z

**Published:** 2023-10-02

**Authors:** Inmaculada Moreno-Gázquez, Raquel Pérez-Palacios, Lucia Abengochea-Quílez, Carmen Lahuerta Pueyo, Ana Roteta Unceta Barrenechea, Alejandro Andrés Gracia, Miguel Angel Aibar Arregui, Sebastián Menao Guillén

**Affiliations:** 1 Department of Clinical Biochemistry, Hospital Clínico Universitario Lozano Blesa, Zaragoza, Spain; 2 Department of Anatomy, Embryology and Genetics, Veterinary Faculty, University of Zaragoza, Zaragoza, Spain; 3 Health Research Institute in Aragón, Zaragoza, Spain; 4 Department of Nuclear Medicine, Multihospital Nuclear Medicine Clinical Unit of Aragón, Zaragoza, Spain; 5 Department of Internal Medicine, Hospital Clínico Universitario Lozano Blesa, Zaragoza, Spain; 6 Department of Chemical and Environmental Engineering, Campus Río Ebro- Edificio I+D, University of Zaragoza, Zaragoza, Spain; 7 Basic Research in Internal Medicine Group, GIIS-084 (IIS Aragón), Zaragoza, Spain

**Keywords:** ATTRwt amyloidosis, Gene variants, Targeted sequencing, Transthyretin amyloidosis

## Abstract

**Objective:**

Wild-type transthyretin (ATTRwt) amyloidosis is caused by the misfolding and deposition of the transthyretin protein (TTR) in the absence of mutations in the *TTR* gene. Studies regarding the variant form of ATTR amyloidosis (ATTRv) suggest that the presence of single-nucleotide polymorphisms (SNP) in genes other than the *TTR*, may influence the development of the disease. However, other genetic factors involved in the aetiopathogenesis of ATTRwt are currently unknown. This work investigates the presence of sequence variants in genes selected for their possible impact on ATTRwt amyloidosis. To do so, targeted sequencing of 84 protein-coding genes was performed in a cohort of 27 patients diagnosed with ATTRwt.

**Results:**

After applying quality and frequency filtering criteria, 72 rare or novel genetic variants were found. Subsequent classification according to the ACMG-AMP criteria resulted in 17 variants classified as of uncertain significance in 14 different genes. To our knowledge, this is the first report associating novel gene variants with ATTRwt amyloidosis. In conclusion, this study provides potential insights into the aetiopathogenesis of ATTRwt amyloidosis by linking novel coding-gene variants with the occurrence of the disease.

**Supplementary Information:**

The online version contains supplementary material available at 10.1186/s13104-023-06491-z.

## Introduction

The misfolding of the transthyretin protein (TTR) leads to its aggregation and systemic extracellular deposition in the form of amyloid fibrils, causing ATTR amyloidosis. TTR misfolding can originate either from inherited mutations in the *TTR* gene, which results in the variant form of the disease (ATTRv), or it can develop spontaneously due to the incorrect folding of the non-mutated TTR, leading to the so-called wild-type ATTR (ATTRwt) amyloidosis. The latter usually affects elderly men and is accompanied by cardiac involvement [1, 2]. Several studies in patients with ATTRv amyloidosis suggest that the presence of single nucleotide polymorphisms (SNPs) in genes other than the *TTR* may influence the development of the disease, affecting its age of onset [3–6]. However, it is currently unknown whether any genetic factors could be involved either in the onset or the development of ATTRwt amyloidosis.

In this work, we study the presence of variants in genes selected for their potential involvement in ATTRwt amyloidosis according to their function. We particularly focused on genes encoding proteases potentially involved in TTR cleavage and their inhibitors, genes encoding TTR-interacting proteins, extracellular chaperones, extracellular matrix (ECM) related proteins, and genes described in the literature to be altered in different types of amyloidosis. Specifically, we designed and performed targeted sequencing of a panel of 84 protein-coding genes in a cohort of patients diagnosed with ATTRwt amyloidosis.

## Main text

### Materials and methods

#### Participants

A total of 27 non-related patients diagnosed with ATTRwt amyloidosis were enrolled in the study. The age of the patients ranged between 68 and 92 years (average age 81 years, 77.8% male). Diagnosis was made following the criteria of Gillmore et al. for cardiac amyloidosis [7] and Sanger sequencing of the *TTR* gene to confirm ATTRwt, at the Hospital Clínico Universitario Lozano Blesa, in Zaragoza, Spain. Clinical features of all patients are summarized in Additional file 1: Table S1.

#### Gene selection and panel design

An Ion AmpliSeq On-Demand panel (Thermo Fisher Scientific) expanded with a spike-in panel was designed with 2 primer pools to sequence 84 genes. Information about the sequencing data produced in this study is available in Additional file 1: Tables S2, S3.

Genes were eligible for inclusion when meeting any of the following criteria:

(i) Being involved in any of the molecular pathways that could play a role in amyloid formation, such as proteolysis, protein folding and ECM maintenance.
(ii) Having SNPs associated in the literature with the age of onset of ATTRv amyloidosis.
(iii) Showing altered expression or methylation patterns in patients with ATTRv amyloidosis.
(iv) Reported as altered in other amyloidotic processes, e.g. Alzheimer’s disease.
(v) Genes coding for proteins that interact with the TTR protein.

#### Genetic analysis

Genomic DNA was isolated from EDTA blood samples using the MagDEA DxSV kit (Precision System Science Co.). DNA libraries were constructed using the Ion AmpliSeq™ Kit, and sequenced on the Ion Chef™ Instrument according to the manufacturer's instructions.

#### Analysis of DNA sequencing data

Reads were aligned to the hg19 human reference genome using the Torrent Suite and IonReporter 5.10.5.0 software.

Filtering criteria for gene variants based on:

1. Reads depth higher than 30.
2. Exonic variants, and intronic variants located up to ± 7bp to the intron–exon boundary.
3. Selection of rare or novel gene variants. Specifically, variants with a minor allele frequency (MAF) ≤ 1% in the Genome Aggregation Database (GnomAD), hence excluding frequent variants in general population with likely no clinical significance.

#### Variant prioritization and classification

Variants were classified and prioritized according to the criteria of the American College of Medical Genetics (ACMG) [8], based on: (i) their location in the DNA sequence; (ii) the variant predicted effect on the protein using variant pathogenicity prediction programs such as SIFT, PolyPhen-2 and MutationTaster; (iii) the predicted effect of the variant on the splicing site, using the splice prediction program Splice AI, (iv) their MAF in GnomAD database; and (v) their presence in the dbSNP polymorphism database and the ClinVar disease database. Variants were also analysed with the Varsome (https://varsome.com/) and Intervar (https://wintervar.wglab.org/) clinical interpretation online software packages.

## Results

After applying the filtering criteria, 72 genetic variants were detected (Additional file 1: Table S4). According to the criteria established by the ACMG [8], 55 of the filtered variants were classified as benign or likely benign. The remaining 17 gene variants, from a total of 14 different genes, were classified as of uncertain significance (VUS). All variants were found in heterozygosis. Detailed information of the identified variants is shown in Table 1.

**Table 1:** Characteristics of variants of uncertain significance found in the study

Functional category	Gene	Sample	dbSNP ID	Transcript	Nucleotide change	Protein change	Allele Frequency in our study	MAFa in GnomAD v2.1.1	ClinVar Classification	SIFT^b^	PolyPhen-2^c^	Mutation Taster^d^	SpliceAI^e^
Altered expression patterns in ATTR amyloidosis	*TF:*	M14	rs121918677	ENST00000402696.3	c.2012G > A	p.Gly671Glu	0.019	0.002491	ID: 12,616. Conflicting interpretations of pathogenicity (Pathogenic/ Likely benign). Atransferrinemia and transferrin variant B2	0,002 (D)	1 (D)	0.999 (D)	–
	*CP*	M14	rs139633388	ENST00000264613.6	c.2684G > C	p.Gly895Ala	0.019	0.001464	ID 42054. Conflicting interpretations. Aceruloplasminemi and deficiency of ferroxidase	0.008 (D)	1 (D)	1 (D)	–
	*CP*	M14	rs202217551	ENST00000264613.6	c.929G > A	p.Arg310His	0.019	0.0002017	ID 546261. Uncertain significance. Deficiency of ferroxidase	0.209 (T)	0.211 (B)	0.995 (D)	–
Altered in other amyloidotic diseases	*ABCA1*	M26	rs140365800	ENST00000374736.3	c.3053A > G	p.Asp1018Gly	0.019	0.0006329	ID 364423. Conflicting interpretations of pathogenicity (VUS or benign). Tangier disease and primary hypoalphalipoproteinemia	0.013 (D)	0.999 (D)	1 (D)	–
	*APP*	M2	rs200857049	ENST00000346798.3	c.446A > G	p.His149Arg	0.019	–	ID: 1,938,835. Uncertain significance. Alzheimer's disease	0.426 (D)	0.943 (D)	1 (D)	–
	*GC*	M24	–	ENST00000504199.1	c.1184G > A	p.Cys395Tyr	0.019	–	–	0.018 (D)	1 (D)	1 (D)	–
Chaperones	*FGA*	M12	rs748106542	ENST00000302053.3	c.514G > A	p.Asp172Asn	0.019	0.00006404	–	0.0 (D)	0.997 (D)	1 (D)	–
	*FGA*	M6	rs150073296	ENST00000302053.3	c.2285C > T	p.Ala762Val	0.019	0.0001698	–	0.001 (D)	0.967 (D)	1 (D)	–
	*FGB*	M20	–	ENST00000302068.4	c.676C > T	p.Pro226Ser	0.019	–	–	0.002 (D)	0.998 (D)	1 (D)	–
ECM components	*ECM1*	M6	rs144607263	ENST00000369047.4	c.1083C > T	p.Ala361 = (splice region)	0.019	0.0000521	–	–	–	1 (D)	0 (Benign)
	*FN1*	M7	–	ENST00000354785.4	c.7304G > A	p.Gly2435Asp	0.019	–	–	0.192 (T)	0.967 (D)	1 (D)	–
	*HSPG2*	M6	–	ENST00000374695.3	c.7795G > A	p.Val2599Met	0.019	–	–	0.019 (D)	0.999 (D)	0.646 (D)	–
	*MMP9*	M16	rs1490219180	ENST00000372330.3	c.1159 T > G	p.Phe387Val	0.019	0.00002804	–	0.003 (D)	0.071 (B)	1 (D)	–
Proteolysis	*F2*	M24	rs760923326	ENST00000311907.5	c.341C > T	p.Thr114Met	0.019	0.000003983	–	0.374 (T)	0.021 (B)	1 (N)	–
	*PLAT*	M16	–	ENST00000220809.4	c.920C > T	p.Pro307Leu	0.019	–	–	0.005 (D)	0.784 (D)	1 (D)	–
	*PLAT*	M26	rs61755432	ENST00000220809.4	c.1481G > C	p.Gly494Ala	0.019	0.0009440	ID: 810,282. Uncertain significance. Thrombocytopenia	0.182 (T)	0.98 (D)	0.999 (D)	–
	*PSEN2*	M12	rs150400387	ENST00000366783.3	c.184C > T	p.Arg62Cys	0.019	0.0002195	ID: 567,646. Alzheimer disease 4 and Dilated cardiomyopathy 1 V	0.012 (D)	0.877 (D)	0.803 (N)	–

^a^*MAF* Minor Allele Frequency

^b^SIFT score: < 0.05 deleterious (D); > 0.05 tolerated (T). For missense variants only

^c^PolyPhen2 score: < 0.15 benign (B); > 0.15 likely pathogenic (D). For missense variants only

^d^MutationTaster score: probability that the prediction is true, higher when close to 1. Likely pathogenic (D); Likely benign (N)

^e^Splice AI score: probability of the variant being splice-altering. From 0 to 1, higher when close to 1

**Fig. 1:**
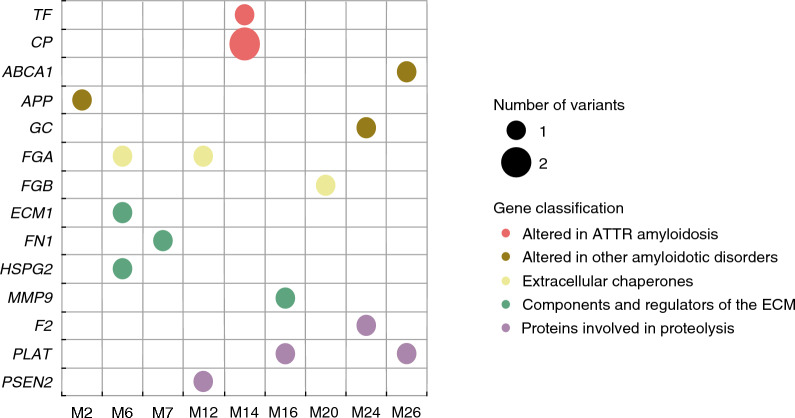
Matrix bubble plot displaying the genes where sequence variants were identified and their corresponding ATTRwt amyloidosis carrier patients (M). Genes are classified by color according to their functional group. *TF* Transferrin; *CP* Ceruloplasmin; *ABCA1* ATP binding cassette subfamily A member 1; *APP* Amyloid beta precursor protein; *GC* GC vitamin D binding protein; *FGA* Fibrinogen Alpha Chain; *FGB* Fibrinogen Beta Chain; *ECM1* Extracellular matrix protein 1; *FN1* Fibronectin 1; *F2* Coagulation factor II—thrombin; *FN1* Fibronectin 1; *HSPG2* Heparan sulfate proteoglycan 2; *MMP9* Matrix metallopeptidase 9; *F2* Coagulation factor II—thrombin; *PLAT* Plasminogen activator—tissue type; *PSEN2* Presenilin 2; *ECM* Extracellular matrix

The identified VUS were distributed among 9 of the subjects under study (Fig. 1) and corresponded to the genes encoding proteases *F2*, *PLAT* and *PSEN2*; the ECM components *ECM1*, *FN1, HSPG2* and *MMP9*; the extracellular chaperones *FGA* and *FGB*; the genes *CP* and *TF,* previously found altered in cardiac ATTR amyloidosis [9, 10]; and the genes *ABCA1, APP* and* GC*, found altered in Alzheimer’s disease [11–13]. Detailed information of the VUS is shown in Table 1. All VUS were missense, excepting one synonymous variant affecting a splice site. While *CP*, *FGA* and *PLAT* genes exhibited 2 different variants each, only one variant was found in the rest of the genes.

All VUS appeared only once in our cohort of patients, being their allele frequency in our sample of 0.019 (Table 1). The variants located in the genes *APP, FGB, FN1, GC, HSPG2* and one of the two located in *PLAT* were novel, being to date absent in the GnomAD database.

## Discussion

In this study we evaluated the presence and predicted pathogenicity of sequence variants in genes that could play a role in the pathogenic mechanisms of ATTRwt amyloidosis. Using a panel of 84 genes selected based on their potential relationship with the disease, we found 72 rare or novel variants associated for the first time with a cohort of ATTRwt amyloidosis patients (Additional file 1: Table S4). Among them, although no pathogenic variants were identified, 17 variants concerning the *ABCA1, APP, CP, ECM1, F2, FGA, FGB, FN1, GC, HSPG2, MMP9*, *PLAT, PSEN2* and *TF* genes were further classified as VUS (Table 1).

A variant is classified as VUS when it does not fulfil all the criteria to be classified as pathogenic or benign, or because it exhibits conflicting evidence. Consistently, these VUS variants may be reclassified as new evidence on them are provided [8]. Therefore, their pathogenic role cannot be ruled out with the current knowledge, and further investigation would be instrumental to understand their clinical significance and relation to the ATTRwt aetiopathogenesis. Of the 17 VUS identified, 10 have been classified as potentially deleterious by all the variant pathogenicity prediction programs consulted: SIFT, PolyPhen-2 and MutationTaster, with high scores (Table 1).

Apart from the disease-specific peptide, amyloid deposits are composed of general amyloid-associated molecules, such as the serum amyloid P component (SAP), apolipoprotein E or ECM-related proteins [14]. Moreover, the deposition of amyloid takes place in the ECM of tissues and organs.We identified one VUS in the *ECM1* gene, which encodes the extracellular matrix protein 1*;* the *FN1* gene, encoding for fibronectin; the *HSPG2* gene, encoding the heparan sulfate proteoglycan (HSPG) 2 or perlecan; and the *MMP9*, which encodes the matrix metalloproteinase 9. Changes in the expression level of the *ECM1* gene have been associated with various non-amyloidotic cardiac conditions [15, 16]. Regarding amyloidosis, Misumi et al. [17] observed that amyloid deposition of TTR induced an increased expression of ECM components including fibronectin in patients with ATTRV30M amyloidosis. An increased expression of *MMP9*, a previously known remodeler of the ECM [18] with capacity to degrade TTR aggregates and fibrils in vitro in the absence of SAP [14] has been also reported in both animal models and patients with ATTRV30M [14, 17, 19]. Interestingly, ECM1 has been shown to interact with MMP9 and to reduce the proteolytic activity of MMP9 in vitro [20]*,* potentially being able to have an indirect effect on the deposits of TTR fibrils. HSPGs are glycoproteins found as components of amyloid deposits in most amyloidosis [21]*.* The highly sulfated domains of heparan sulfate and its analogue heparin interact with V30M TTR and recombinant TTRwt, and have been suggested to function as a scaffold for TTR fibril formation in ATTR amyloidosis and to foster TTR fibrillization [22, 23]. Interestingly, the selective heparin/heparan sulfate-TTR binding was stronger for the TTRwt peptide than for the equivalent carrying the V30M mutation [23]. Hence, the presence of a variant in the ECM proteins identified here as VUS, could induce changes in the binding or function activities of such proteins and eventually influence the formation of TTRwt amyloid fibrils.

*A*myloid deposits can be formed either by the complete TTR protein or TTR fragments [24, 25]. Some studies attribute TTR cleavage to trypsin-like serine proteases [26]. In our data, we found VUS in 3 genes encoding proteases: *PLAT* gene, encoding tissue plasminogen activator, *F2* gene, encoding prothrombin, and *PSEN2* gene, encoding Presenilin 2. Blood concentration of prothrombin has been reported lower in patients with ATTRwt amyloidosis in comparison to healthy controls and individuals with ATTRv amyloidosis [9]. Regarding Presenilin-2, it is part of the complex that catalyzes the cleavage of APP and mutations in *PSEN2* are causative of dominantly inherited Alzheimer’s disease [27].

Extracellular chaperones stabilize misfolded proteins and guide them to specific cell receptors for their uptake and subsequent degradation. It has been suggested that their dysfunction may result in the deposition of misfolded proteins, influencing the development of amyloidosis disorders [28]. In this study, we identified 2 VUS in the *FGA* gene, and 1 in the *FGB*, coding for the alpha and beta chain of fibrinogen, respectively. Da Costa et al. [29] reported increased plasma concentrations of fibrinogen and other extracellular chaperones in patients with ATTRV30M. Concentrations of the alpha chain of fibrinogen were also higher in patients with ATTRv amyloidosis when compared to the ATTRwt amyloidosis cohort [9]. In addition, mutations in *FGA* are associated with fibrinogen A α-chain amyloidosis, a type of hereditary renal amyloidosis [30].

It is also conceivable that there may be shared alterations with other amyloidotic disorders, such as Alzheimer's disease. We found VUS in the *ABCA1, APP* and *GC* genes, the three reported as altered in Alzheimer’s disease [13, 31]. An impaired cleavage of APP protein results in the generation and extracellular secretion of β-amyloid that will ultimately assemble to form amyloid plaques [32]. Approximately 25 pathogenic variants in the *APP* gene have been associated to the etiology of Alzheimer’s disease [12]. Curiously, it has been proposed that APPwt could function as a transcriptional regulator of TTRwt [33, 34]. Moreover, TTR deposits can co-exist with β-amyloid ones in patients with ATTRv amyloidosis [35].

Finally, we identified VUS in genes for which alterations have been reported in the literature in patients with ATTRwt amyloidosis, as it is the case of the *CP* gene, responsible for ceruloplasmin, and *TF* gene, coding for transferrin [9]. CP is increased in patients with ATTRwt amyloidosis relative to healthy controls [9]. For their part, Ohta et al. [10] reported the co-precipitation of transferrin with TTR amyloid in mouse models with TTRV30M and suggested that transferrin facilitates the destabilization of the secondary structure of TTR, contributing to fibrillogenesis.

In summary, this study provides novel data about the identification of variants in diverse protein-coding genes that we hypothesize might not be disease-causing, but do rather modulate or contribute to the development of ATTRwt amyloidosis. Although some of the selected genes have been previously assessed in ATTRv amyloidosis, this is the first time that variants in such genes are identified in patients with ATTRwt amyloidosis.

To conclude, our work can serve as a basis for future studies focused on unravelling the underlying mechanisms of TTR misfolding and expand the understanding of the disease.

## Limitations

The present study has several limitations: (i) the size of our study cohort was small, (ii) the described variants should be further examined in non-disease-affected relatives of the patients under study, and (iii) the number of genes that could be included in the sequencing panel was limited. Thus, further investigation of the newly identified variants and the broadening of the targeted genes should be pursued in future studies.

### Supplementary Information

**Additional file 1:** **Table S1.** Clinical features of the patients included in the study. **Table S2.** Genes included in the On-Demand panel, number of amplicons, number of bases and coverage. Genes are grouped by functional category. **Table S3.** Genes included in the Spike-in panel, number of amplicons, number of bases and coverage. Genes are grouped by functional category. **Table S4.** Characteristics of all variants found in the study.

## Data Availability

The data generated from this study (list of identified variants) are available in Additional file 1: Table S4. Information of variants identified are openly available online at ClinVar (https://www.ncbi.nlm.nih.gov/clinvar/), dbSNP (https://www.ncbi.nlm.nih.gov/snp/) and gnomAD (http://gnomad.broadinstitute.org/) databases.

## Abbreviations

TTR: Transthyretin
ATTRwt: Wild-type ATTR amyloidosis
ATTRv: Variant ATTR amyloidosis
SNPs: Single nucleotide polymorphisms
ECM: Extracellular matrix
MAF: Minor allele frequency
GnomAD: Genome aggregation database
ACMG: American College of Medical Genetics
VUS: Variant of uncertain significance
APP: β-Amyloid precursor protein
